# Polycythemia vera and essential thrombocythemia in children, still a challenge for pediatricians

**DOI:** 10.1007/s00431-025-05993-1

**Published:** 2025-02-04

**Authors:** Agathe Picard, Sophie Bayart, Marianna Deparis, Cécile Dumesnil De Maricourt, Sophie Haro, Anne Jourdain, Coralie Mallebranche, Fanny Rialland, Damien Luque Paz, Cedric Pastoret, Virginie Gandemer, Elie Cousin

**Affiliations:** 1 Pediatric Oncohematology Department, Rennes University Hospital, Rennes, France; 2 Pediatric Oncohematology Department, Caen-Normandie University Hospital, Caen, France; 3 Pediatric Oncohematology Department, Rouen University Hospital, Rouen, France; 4 Pediatric Oncohematology Department, Brest University Hospital, Brest, France; 5 Pediatric Oncohematology Department, Tours University Hospital, Tours, France; 6 Pediatric Oncohematology Department, Angers University Hospital, INSERM, CRCI2NA, SFR ICAT, Angers, Nantes University, Nantes, France; 7 Pediatric Oncohematology Department, Nantes University Hospital, Nantes, France; 8 Univ Angers, Nantes Université, CHU Angers, Inserm, CNRS, CRCI2NA, F-49000 Angers, France; 9 Laboratory of Hematology Department, Rennes University Hospital, Rennes, France

**Keywords:** Myeloproliferative neoplasms, Polycythemia vera, Essential thrombocythemia, Pediatrics, Adolescents and young adults, Follow-up

## Abstract

Polycythemia vera (PV) and essential thrombocythemia (ET) are rare myeloproliferative neoplasms (MPN) in children, adolescents and young adults. No recommendations are available concerning these patients’ management. Transposing to children the knowledge established in adult patients is not acceptable. For a better understanding of difficulties encountered by pediatricians and adult hematologists, we conducted a national practice analysis concerning follow-up of patients under 18 diagnosed with ET or PV, in France. Then, we present data from a multicentric, descriptive, retrospective study, including 17 patients with ET or PV, diagnosed under 18, coming from 7 hematopediatric departments in France. Interviewed physicians reported a lack of expertise and theoretical training in the hematological field to diagnose and follow children with MPNs. Data from 17 patients (15 ET, 2 PV) confirmed a high proportion of asymptomatic patients at the time of diagnosis (41%). Proportion of “triple-negative” patients (59%) was higher than in adult cohorts. 60% of patients underwent a bone marrow biopsy and 31% of cases were discussed during a multi-disciplinary staff meeting. 76.5% patients were treated, with a high frequency of antithrombotic and cytoreductive drugs. No complications were observed during the 45 months of median follow-up. *Conclusion*: Physicians insisted on the need for training. Only the accumulation of descriptions of MPNs in children will lead to a better management of these diseases. Considering the small proportion of pediatric patients with complications after diagnosis, rapid therapeutic de-escalation seems essential to consider during the follow-up in a close collaboration with adult hematologists.

**Table:** 

**What is Known:** *• Myeloproliferativ neoplams are rare and chronic deseases, most of the time affecting adults but also found in few pediatric patients.* • *There are no recommendations for the diagnosis, therapeutic management or follow-up of children with polycythemia vera or essential thrombocythemia*.	
**What is New:** *• Find out how adult haematologists and paediatricians feel about the management and follow-up of paediatric patients with myeloproliferative syndrome through a national practice analysis.* • *Description of the “real life” follow-up of children with polycythemia vera or essential thrombocythemia in France*.

**Supplementary Information:**

The online version contains supplementary material available at 10.1007/s00431-025-05993-1.

## Introduction

Polycythemia vera (PV) and essential thrombocythemia (ET) are rare myeloproliferative neoplasms (MPNs) in children, adolescents, and young adults (AYA). The median age at diagnosis for these diseases is typically over 60 years [3]. MPNs are caused by somatic mutations in driver genes such as JAK2, MPL, or CALR, which are present in approximately 80% of adult cases but occur less frequently in children [1–4]. A recent review by Ianotto et al. analyzed the characteristics and outcomes of PV and ET in children and AYA, highlighting the limited published data on these conditions. According to the review, the global incidence of MPNs in children and AYA is estimated to be around 0.82 per 100,000 patients per year, with the incidence of ET and PV being approximately 0.6 and 0.18 per 100,000 patients per year, respectively [5].

Due to the rarity of PV and ET in pediatric patients, pediatricians may be unfamiliar with MPNs. Early diagnosis of these conditions is necessary for referring patients to specialized hematology units and determining the need for specific treatments to prevent long-term complications such as thrombosis, hemorrhage, or progression to acute leukemia or secondary myelofibrosis. As cytoreductive drugs can carry toxicities, particularly when initiated at a young age, clear treatment guidelines and long-term follow-up are essential. Pediatricians, including hematology specialists, may hesitate to manage these patients due to limited experience, while adult hematologists may face challenges related to the unique psychological, social, and educational aspects of managing pediatric patients. The development of specialized AYA units has greatly improved oncology care for this age group, but data on MPNs in this population remain scarce. To better understand the challenges faced by healthcare providers, we present the findings of a national practice analysis on the follow-up of children and AYAs diagnosed under 18 with MPNs in France. Additionally, we report the real-world clinical, biological, and follow-up data from 17 pediatric patients with ET or PV, drawn from seven pediatric hematology departments in western France.

## Material and methods

First, a national survey was conducted. It concerned the follow-up of children and AYA diagnosed with ET or PV before 18 years old, in France. A survey (*SurveyMonkey*Ⓡ *application*) of 8 questions (annex 1) was sent by email to all pediatrician members of the leukemia committee of *Société Française de lutte contre les Cancers et leucémies de l’Enfant et de l’adolescent (SFCE)*, and all hematologist members of *France Intergroupe des Syndromes Myéloprolifératifs* (*FIM*). The survey was sent out in October 2022 and closed after two reminders.

In the second part, we report real-life data of multicentric retrospective study including clinical, biological, genetic, and follow-up features of 17 ET or PV pediatric patients, coming from 7 pediatric hematology departments, belonging to the *GOCE* group (*Grand Ouest Cancer de l’enfant*), including French university hospitals of Rennes, Angers, Nantes, Brest, Caen, Rouen, and Tours. Data concerning consultation frequency was collected. Consultation dates were recorded from diagnosis until the latest follow-up. Patients diagnosed with PV or ET before 18 in the *GOCE* group were eligible for inclusion. Diagnosis was left to the discretion of clinicians. Eligible patients were identified by clinicians and biologists from each local pediatric hematological department. Inclusions took place between December 2023 and September 2024. Protocols and patient information letters were approved by the Ethical Committee of Rennes University Hospital.

## Results

1. National practice analysis

**Fig. 1:**
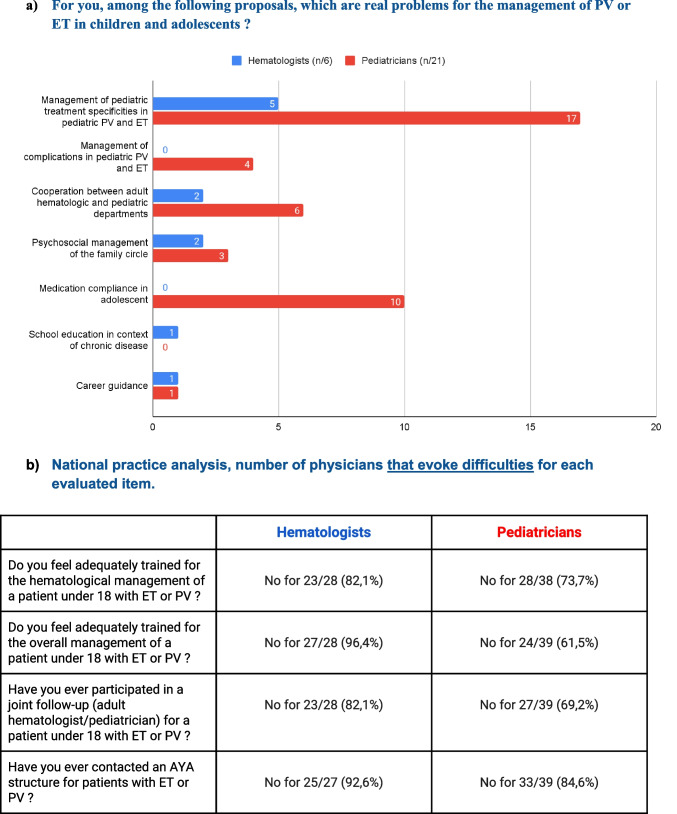

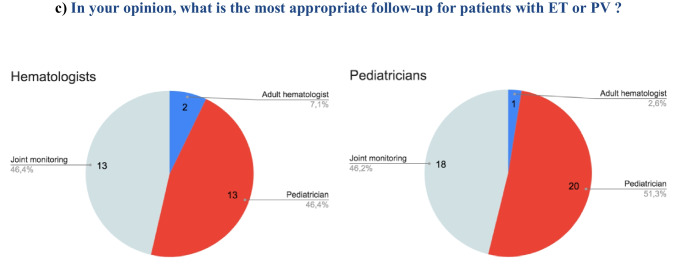
Results of the national practice analysis survey

Forty answers were collected among the pediatrician members of the leukemia committee of *SFCE*, and 28 answers among the hematologists, members of *FIM*. Detailed results can be found in Fig. 1. First, we noticed that among pediatric experts, only 20/40 (50%) have already followed a patient under 18 with a PV or ET, and 5/28 (18%) among hematologists.

To present the survey’s results, we reported the number of physicians that highlighted difficulties for each evaluated item (Fig. 1a). The main issue reported by physicians, related to pediatric patients’ follow-up, was management of specific hematological treatments: 81% of pediatricians, 83% of hematologists. Among other evaluated issues, the following items seemed to raise fewer problems: ability to manage specific disease complications, cooperation between hematologic and pediatric departments, psychological management of family circle, school education and management of career guidance. Management of medication compliance remained hazardous for half of pediatricians.

Both interviewed groups reported the need for expertise and theoretical training in the hematological field to safely follow children affected by ET or PV, and to ensure adequate global care (Fig. 1b).

Majority of interviewed participants considered that children with ET and PV should be followed by pediatricians (Fig. 1c). Joint monitoring seemed to be a right choice to follow children with ET and PV but only few interviewed physicians experienced joint consultation (Fig. 1b). In case of joint monitoring, the majority of participants did not encounter difficulties with cooperation between units (9/14 (64%) of pediatricians, 5/6 (83%) of hematologists). This survey also told us that specialized AYA units were hardly ever solicited for adolescents with ET and PV (Fig. 1b).

2) Retrospective study data

Clinical, biological, genetic data, treatment and complications features of 17 ET (15/17) and PV (2/17) patients, diagnosed before 18, coming from 7 pediatric oncohematology departments located in the west part of France, were collected and studied. Since only two pediatric patients had PV, epidemiological data have been pooled. See details in Table 1.

**Table 1:** Clinical, biological, genetic features, treatments, and complications for patients with ET and PV diagnosed before 18, coming from 7 pediatric oncohematology departments located in the west part of France

Number total of cases—ET/PV	17–15/2
Median age at diagnosis, years (range, min–max)	7.69 (0.11–16.67)
Sex ratio (m/f)	6/11
Symptoms at diagnosis, *n*/total (%)	
Asymptomatic	7/17 (41.1)
Thrombosis	3/17 (17.6)
Hemorrhage	0
Splenomegaly	3/15 (20)
Headache	6/17 (35.3)
Bone pain	3/17 (17.6)
Paresthesia/erythromelalgia	1/17 (5.9)
Fatigue	2/17 (11.8)
Pruritus	2/17 (11.8)
Full blood count at diagnosis ET/PV*	
Leukocytes (G/L)	10.26/8.59
Platelets (G/L)	1078.5 / 574
Hemoglobin (g/dL)	12.61 / 16.15
Hematocrit (%)	37.02 / 51.1
Anatomo-pathology, *n*/total (%)	
Bone marrow biopsy	9/15 (60)
Driver mutations, *n*/total (%)	
*JAK 2 exon 14*	6/17 (35.3)
Allele burden % mean (ET%/PV%)	11.58/37.9
*CALR*	1/17 (5.9)
*MPL*	0
Triple negative (for *JAK2*, *CAL-R* et *MPL*)	10/17 (58.8)
Medical treatment, *n*/total (%)	
Yes, *n*/17 (%)	13/17 (76.5)
Antithrombotic treatments, *n*/total (%)	
No antithrombotic treatment	8/17 (47)
Low-dose aspirin	7/17 (41.2)
Rivaroxaban	1/17 (5.9)
Apixaban	1/17 (5.9)
Cytoreductive drugs, *n*/total (%)	
No cytoreductive drug	7/17 (41.2)
Hydroxycarbamide	7/17 (41.2)
Interferon	4/17 (23.5)
Venesection	1/17 (5.9)
Anagrélide	2/17 (11.8)
Ruxolitinib	1/17 (5.9)
Complications after diagnosis, *n*/total (%)	
Hemorrhage	1/17 (5.9)
Thrombosis	0
Acute leukemia	0
Myelofibrosis	0
Treatment toxicities	3/17 (17.6)
Follow-up, *n*/total (%)	
Median follow-up (months)	45
Multi-disciplinary staff meeting	5/16 (31)
Transition consultation from pediatric to adult healthcare	4/10 (28.6)

^*^Mean values

### Clinical characteristics

Median age at diagnosis was 7.69 years old, with a sex ratio (m/f) of 1/2. At the time of diagnosis, 7/17 (41%) patients were asymptomatic; then, diagnosis was based on blood counts abnormalities discovered incidentally (biology taken routinely or for symptoms not related to MPNs). The most frequent symptom experienced was headache. Three patients (2 PV and 1 ET) were diagnosed following a thrombotic event and were then considered “high-risk” patients: a young girl was diagnosed with PV in the context of a portal thrombosis with esophageal varices, a second patient with PV was diagnosed after a cerebral venous sinus thrombosis associated with portal and splanchnic thrombosis, and a young boy was diagnosed with ET following a transient ischemic attack. In our study, only 3 patients (3/15; 20%) had palpable spleen at time of diagnosis.

### Biological characteristics

All biological data are detailed in Table 1. For ET patients, the mean platelet count was 1078 G/L, the mean hemoglobin concentration was 12.6 g/dL. For PV patients, the mean hemoglobin concentration was 16.15 g/L, the mean hematocrit was 51.1%, and the mean platelet count was 574 G/L. In this cohort, bone marrow biopsy has been performed in 9/15 patients (60%). In the other cases, diagnosis was based on bone marrow smear and gene mutations’ detection.

### Molecular analyses

All patients had a molecular biology panel exploring *JAK2*, *MPL*, and *CALR* mutations, 35% of patients (6/17) were positive for *JAK2 V617F* mutation. The two patients with a diagnosis of PV presented a *JAK2 V617F* mutation. One patient with ET had a *CALR* mutation (type 1, del52pb). Consequently, the percentage of patients without identifiable mutation of either *JAK2* or *CALR* or *MPL* (triple negative) was high: 10/17 (59%). *JAK2* V617F mean allele burden for ET and PV patients were respectively 11.5% and 38%. Next-generation sequencing (NGS), exploring non-driver mutations, was not routinely performed in our population. Two patients with triple negative MPN underwent NGS, where no additional mutation was found.

### Treatment

In our cohort, therapy for 5 out of 16 (31%) patients was determined during a multi-disciplinary staff meeting, with all of them being prescribed medical treatment. Overall, 13 out of 17 (76.5%) patients received treatment for their MPN. Bloodletting was performed at diagnosis for one patient with PV who presented with significant hyperviscosity symptoms. Antithrombotic drugs were frequently prescribed, with 9 out of 17 (53%) patients receiving them initially. More than half of the patients (10/17, 59%) were treated with a cytoreductive drug or related therapy. The use of pegylated interferon was less common (4/17, 23.5%) compared to hydroxyurea, which was prescribed to 7 out of 17 (41%) patients. Only one patient received JAK2 inhibitors due to interferon-related toxicity. The two patients with PV were treated with direct oral anticoagulants (Rivaroxaban, Apixaban) for massive venous thrombosis at diagnosis. As of the last follow-up, 9 out of 17 (53%) patients remain on treatment.

### Follow-up, outcomes and complications

**Fig. 2:**
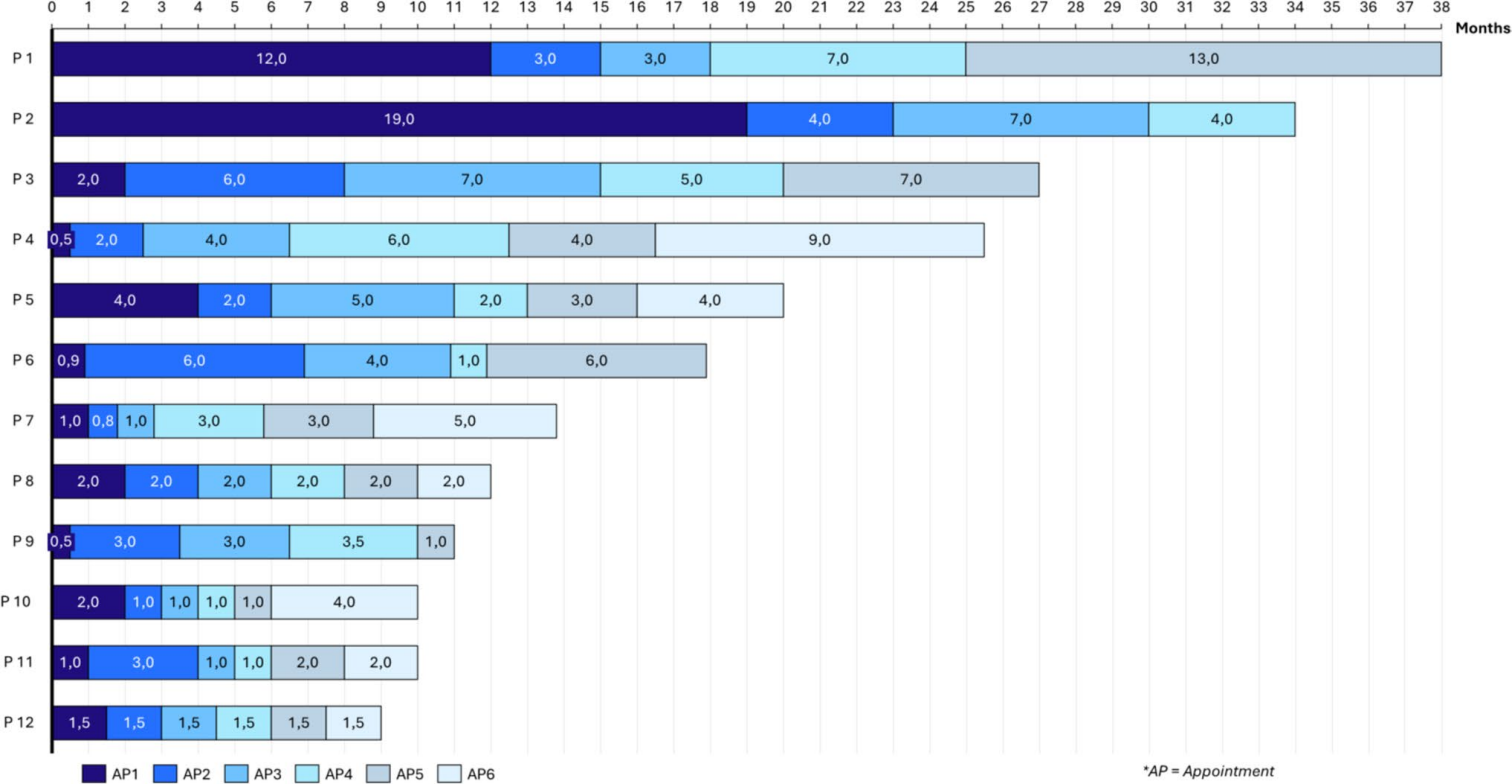
Time between two consultations from diagnosis

The majority of patients were initially followed by a pediatrician. Four patients, all young adults (aged over 18 at the time of inclusion, 10/17), underwent a transition consultation from pediatric to adult healthcare. However, none of the patients were followed by specialized AYA units. There was considerable variability in the frequency of follow-up among the patients, with the time between diagnosis and the first follow-up consultation ranging from 13 days to 19 months. Figure 2 illustrates the intervals between the first six consultations following diagnosis for 12 of the patients.

No patients experienced thrombotic complications or transformations (acute leukemia or secondary myelofibrosis) during the follow-up period (excluding those present at diagnosis). One patient with PV required endoscopy for esophageal variceal ligation due to portal thrombosis at diagnosis. One untreated ET patient had recurrent epistaxis. Notably, two patients experienced toxicities related to hydroxyurea treatment (dyskalemia and anemia, grade 2), and one patient had complications from pegylated interferon (hepatic cytolysis, grade 3; abdominal pain, grade 2, according to the Common Terminology Criteria for Adverse Events). The median follow-up duration was 45 months, and all patients were alive at the end of the follow-up period.

## Discussion

The analysis of practices revealed that only a few physicians, even those with expertise in pediatric hematology, have encountered children or adolescents with MPNs. The primary issue identified was the adaptation of MPN-specific treatments. Since MPNs are rare in children and clinical trials often do not include patients under 18, diagnostic and prognostic criteria have never been validated in the pediatric population, making the management of MPNs in young patients particularly challenging. In this study, the medical management of ET and PV varied significantly between centers and practitioners. A notable heterogeneity in the care provided was observed, particularly in terms of consultation frequency (Fig. 2). Physicians emphasized the need for training, but it is easy to see that shared learning and stronger collaboration between adult hematologists and pediatric experts could help practitioners manage young patients with ET and PV more effectively [6, 7].

**Fig. 3:**
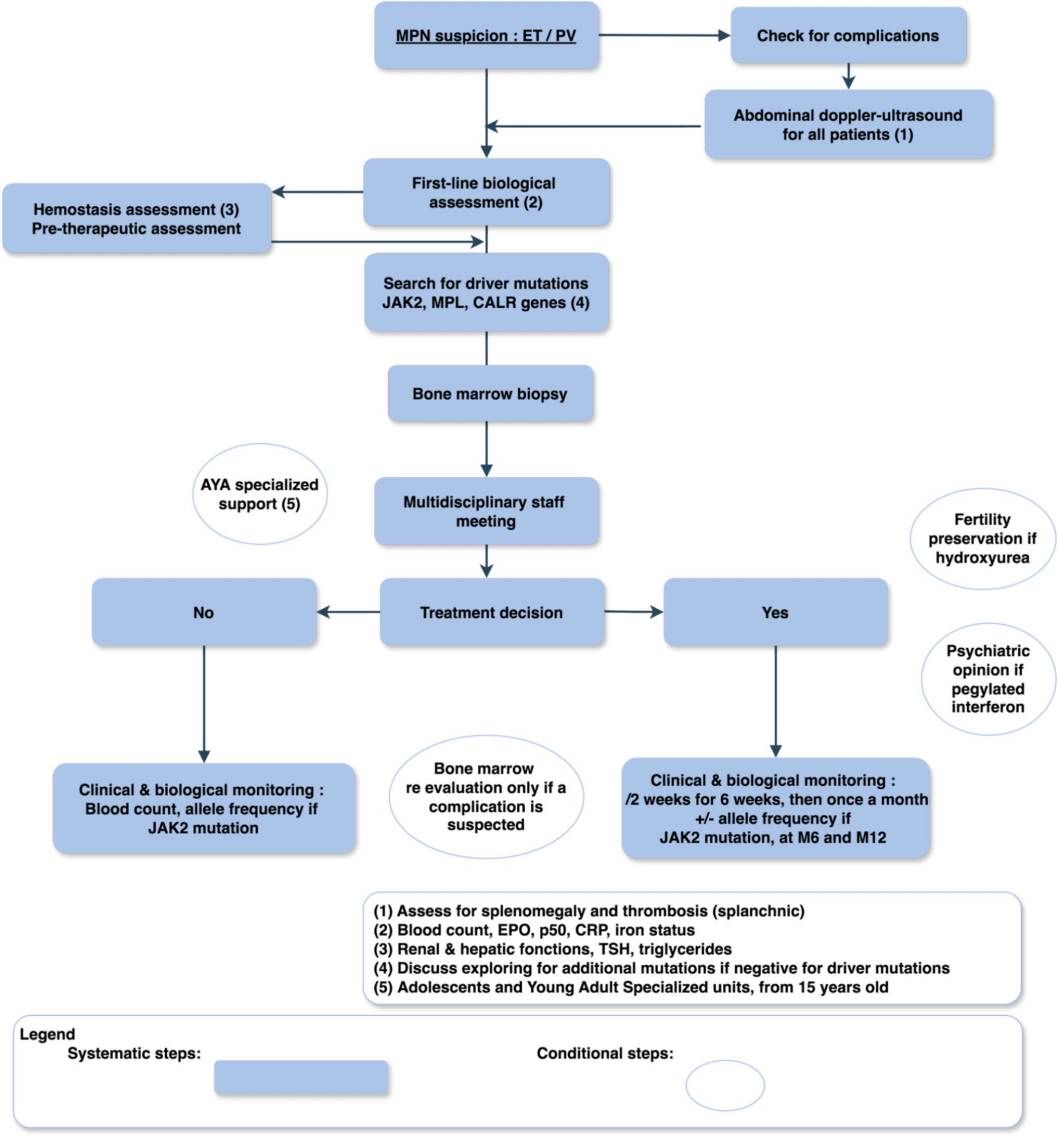
Proposal for an approach to manage pediatric MPNs

In our multicentric retrospective study, the median age at diagnosis was slightly younger than in previous studies [5, 8]. Almost half of children were asymptomatic at time of diagnosis, and the proportion of so-called triple negative patients was near to 50% as described in the literature [5, 8–10]. Without specific clinical symptoms for MPNs, diagnosis was made by chance in almost half of patients, so diagnostic delay was inestimable. Literature indicates that the majority of adult MPNs are associated with somatic mutations that lead to the constitutive activation of the JAK2-STAT5 pathway, with *JAK2 V617F* being the most common mutation. This mutation is found in ≥ 95% of PV cases and approximately 60% of ET cases [2–4, 11]. In line with existing pediatric literature, we observed a lower percentage of *JAK2 V617F*-positive patients [5, 8, 9], 35% (6/17), with variable allelic frequencies (ranging from 3.44 to 51.4%) in ET and PV, respectively [5, 8, 9]. Distinguishing between PV and JAK2-mutated ET can be challenging in pediatric cases [12]. Although histological analysis remains a key criterion for MPN diagnosis according to the WHO, only 60% of our French cohort underwent a bone marrow biopsy at diagnosis. Putti et al. highlighted the critical role of bone marrow biopsy in accurately diagnosing MPNs and ruling out other causes of thrombocytosis. Clinical and biological characteristics can overlap, and driver mutations are rarely found in this age group. In 2019, Kucine et al*.* proposed pediatric MPN diagnostic criteria based on the WHO classification, in which bone marrow biopsy remains a crucial element [12]. Based on these findings, we recommend that all patients under 18 with suspected ET or PV undergo bone marrow biopsy and driver mutation analysis. If no driver mutation is identified or if there is a family history of MPN, additional mutations should be explored through next-generation sequencing (NGS). The NGS panel should include all coding exons of the JAK2 and MPL genes, as rare non-canonical driver mutations can be detected in both MPN and constitutional thrombocythemia/polycythemia [13]. A proposed medical approach for pediatric MPNs is illustrated in Fig. 3.

With a median follow-up of 45 months, no patient presented thrombotic complication (not taking into account patients with complications before diagnosis) and only one patient presented with recurrent epistaxis. On top of predominance for thrombotic venous events, Ianotto et al. [5] reported that the incidence of thrombosis during the follow-up was 9.3% for patients with PV, and 3.8% for those with ET. In the context of these chronic diseases occurring in young patients, only very prolonged follow-up (several decades) could enable the risks of complications to be established more precisely.

The treatment goals in pediatric MPN are to avoid thrombosis and bleeding, treat MPN-related symptoms, and minimize risk of malignant transformation. A relatively high frequency of initial prescription of “antithrombotic” drugs was observed despite the low number of high-risk patients, as defined on history of thrombosis and platelet count (over 1000 G/L) [4, 11, 14] and more than 50% of patients have been treated with a cytoreductive drug or related therapy (10/17, 58.8%). The reasons for prescribing these treatments were not explained. The hypotheses explaining these data could be: the unacceptable nature of persistent symptoms, even moderate in young patients, the apprehension of professionals regarding the risk of transformation, parent’s opinion… This study aims to better characterize the therapeutic indications in the future. Thrombocytosis > 1500 G/L or high elevation of hematocrit (teenage boy > 54%, teenage girl > 47%) [4] are proposed as an indication for treatment by European LeukemiaNet (ELN) but there is no data on a specific threshold for children and AYA. On top of literature, this study highlights that specific treatment such as antithrombotic and cytoreductive drugs (pegylated interferon) should be introduced only if the patient reports specific disabling symptoms, or suffers from complications (thrombosis or hemorrhage). Therefore, extreme thrombocytosis by itself, must not be seen as a formal therapeutic indication in young patients. Initiating a treatment has to be associated with specific precautions such as psychological evaluation before pegylated interferon, or fertility preservation before hydroxyurea. Because ET and PV are very rare conditions in the pediatric population, all newly diagnosed young patients should benefit from a multi-disciplinary meeting, gathering adult hematologists with expertise in looking after MPN and pediatricians to decide the best management and therapy. Our study is in line with relative literature and shows that complications in young patients are rare, and transformation is exceptional. In this case, it is essential to reassess therapeutic indications and discuss therapeutic de-escalations regularly.

In our population, the use of pegylated interferon seemed to be uncommon, compared to the first-line prescription of hydroxyurea. Hydroxyurea is effective in preventing thrombosis for high-risk patients with essential thrombocythemia [15] but could be associated with specific toxicities such as gonadotoxicity, acute leukemia, and myelofibrosis, with particular concern in young patients [12, 16, 17]. Interferon is known as a safe option in young patients and improves myelofibrosis-free survival [17]. Moreover, pegylated interferon has been reported to achieve a better molecular response compared to hydroxyurea. Pegylated interferon would therefore appear to be more appropriate as a first-line treatment for children and adolescents. Ruxolitinib is a JAK2 inhibitor approved for the treatment of PV in adult patients with intolerance or resistance to hydroxyurea treatment, regardless of *JAK2 V617F* mutational status, with well-known efficacy for splenomegaly, potentially reduction of vascular events, and complete molecular remission [18]. The use of ruxolitinib is well tolerated in the adult population, although side effects such as myelosuppression, hyperlipidemia, and risk of viral infections require surveillance and proactive preventive measures [19, 20]. In our cohort, only one patient with PV has been treated by JAK2 inhibitors after a decision in a multi-disciplinary staff meeting. No complication linked to PV or to the treatment was observed. Studies assessing ruxolitinib as treatment for intolerant or resistant children to interferon are needed.

Data concerning follow-up in our study revealed a great heterogeneity. Regular check-ups are required for young patients with ET or PV. Patients must also be warned about lifestyle risks, for example thrombohemorrhagic risks associated with smoking or hormonal contraception. Women with a child desire should be referred to a specialist and benefit from a specific follow-up. Resolution of symptoms, blood count normalization, toxicity, and complications should be followed for treated patients. Mutated allele burden has been validated as a prognosis factor in MPNs. Monitoring of *JAK2*, *CALR*, or *MPL* allele burden has been validated in several protocols following interferon, ruxolitinib, or stem cell transplant [18, 21]. In adult populations, a reduction in the mutated allele burden has been observed in response to various therapies, including interferon, JAK inhibitors, and stem cell transplantation. Although hydroxyurea fails to significantly reduce the JAK2 V617F allele burden, interferon has demonstrated the ability to achieve a sustained molecular response in patients with PV and ET [22]. Minimal residual disease or measurable residual disease (MRD) uses sensitive laboratory methods to find remaining abnormal cells among millions of normal cells after treatment. Initial studies on MRD assessment following JAK inhibitor therapy have reported modest reductions in mutational burden, despite significant improvements in patient survival in myelofibrosis [23]. However, growing evidence suggests that long-term therapy may lead to slow but complete molecular responses in some patients, underscoring the importance of extended MRD monitoring [24]. Thus, MRD evaluation could also be considered and linked to clinical evolution in pediatric cases of JAK2-mutated myeloproliferative neoplasms. Whereas the use of MRD in treatment adaptation needs to be further investigated, we propose monitoring variant allele frequency (VAF) in JAK2 V617F-mutated patients with sensitive techniques such as real-time quantitative PCR or digital PCR, when feasible. Controls of bone marrow smear or biopsy during follow-up should only be carried out in case of further disturbance of blood counts or new clinical symptoms (Fig. 3).

A diagnosis of a chronic disease at the age of 65 years carries a different significance than one made under the age of 18 years or younger, which is why care for young patients must be tailored to their unique needs. Attention must be given to the psychological well-being of both the patient and their family, medication adherence, as well as educational and career guidance in the context of living with a chronic disease. To improve care and follow-up for young patients, studies evaluating quality of life are essential and will be a key part of the ongoing research in this field. The development of specialized AYA units has significantly improved care for this age group in the context of other chronic diseases. Unfortunately, pediatric patients with MPNs do not currently have access to such specialized structures. Therefore, ongoing collaboration between pediatricians and adult hematologists is crucial for optimizing care and management.

## Conclusion

Pediatric MPNs differ from those diagnosed in adults in several key ways. These differences necessitate a care approach that emphasizes collaboration between pediatricians and hematologists. Pediatricians highlight the importance of further training in this area, but the accumulation of case descriptions for PV and ET in children is essential to improving our understanding and management of these rare diseases in young patients. The identification of driver mutations in JAK2, CALR, and MPL genes, along with histological analysis of bone marrow biopsies and multi-disciplinary team meetings, should be standardized for all young patients with ET or PV. If cytoreductive treatment is required, interferon should be the first-line therapy, and regular discussions about therapeutic de-escalation should be a part of the treatment process. Transition consultations between pediatric and adult care teams should follow models used for other chronic diseases. AYA should be referred to specialized units that consider the social, psychological, and educational needs of these patients.

## Supplementary Information

Below is the link to the electronic supplementary material.

Supplementary file1 (DOCX 8 KB)

## Abbreviations

AYA: Adolescents and young adults
BC: Blood count
ELN: European LeukemiaNet
ET: Essential thrombocythemia
FIM: *France Intergroupe syndromes Myéloprolifératifs*
GOCE: *Grand Ouest Cancer de l’Enfan*T
MPN: Myeloproliferative neoplasm
MRD: Minimal residual disease
NGS: Next-generation sequencing
PV: Polycythemia vera
SFCE: *Société Française de lutte contre les Cancers et leucémies de l’Enfant et de l’adolescen*T
VAF: Variant allele frequency
WHO: World Health Organization
