# Supplementary material for: Polycythemia vera and essential thrombocythemia in children, still a challenge for pediatricians
Source: Eur J Pediatr. 2025 Feb 4;184(2):173. doi: 10.1007/s00431-025-05993-1 (PMC11794414; doi:10.1007/s00431-025-05993-1)
Supplement: Supplementary file 1 — Supplementary file1 (DOCX 8 KB) [file 431_2025_5993_MOESM1_ESM.docx]

Annex 1: National practice analysis survey

1. **Do you follow or have you followed adolescents under the age of 18 suffering from polycythemia vera or essential thrombocythemia?**

Yes or No

1. **For you, among the following proposals, which are real problems for the management of PV or ET in children and adolescents ?**

- Management of pediatric treatment specificities in pediatric PV and TE,
- Management of complications in pediatric PV en TE,
- Cooperation between adult hematologic departments and pediatric departments,
- Psychosocial management of the family circle,
- Medication compliance in adolescent,
- School education in context of chronic disease,
- Career guidance.

1. **Do you feel adequately trained for the hematological management of a patient under 18 with ET or PV ?**

Yes or No

1. **Do you feel adequately trained for the overall management of a patient under 18 with ET or PV ?**

Yes or No

1. **In your opinion, what is the most appropriate follow-up for patients with ET or PV ?**

- Adult hematologist,
- Pediatrician,
- Joint monitoring (adult hematologist/pediatrician).

1. **Have you ever participated in a joint follow-up (adult hematologist/pediatrician) for a patient under 18 with ET or PV ?**

Yes or No

1. **If so, was this monitoring easy to set up ?**

Yes or No

1. **Have you ever contacted an AYA (adolescent and young adult) structure for patients with ET or PV ?**

Yes or No
